# Circulation of Enterovirus D68 during Period of Increased Influenza-Like Illness, Maryland, USA, 2021

**DOI:** 10.3201/eid2807.212603

**Published:** 2022-07

**Authors:** Amary Fall, Nicholas Gallagher, C. Paul Morris, Julie M. Norton, Andrew Pekosz, Eili Klein, Heba H. Mostafa

**Affiliations:** Johns Hopkins School of Medicine, Baltimore, Maryland, USA (A. Fall, N. Gallagher, C.P. Morris, J.M. Norton, A. Pekosz, E. Klein, H.H. Mostafa);; National Institute of Allergy and Infectious Disease, National Institutes of Health, Bethesda, Maryland, USA (C.P. Morris);; Johns Hopkins Bloomberg School of Public Health, Baltimore (A. Pekosz);; Center for Disease Dynamics, Economics, and Policy, Washington, DC, USA (E. Klein)

**Keywords:** enterovirus D68, enterovirus, EV-D68, viruses, respiratory infections, Maryland, United States, influenza-like illness

## Abstract

We report enterovirus D68 circulation in Maryland, USA, during September–October 2021, which was associated with a spike in influenza-like illness. The characterized enterovirus D68 genomes clustered within the B3 subclade that circulated in 2018 in Europe and the United States.

In early July 2021, the United States began to relax COVID-19 infection control measures. As the number of COVID-19 cases began to fall, cases of influenza-like illness (Appendix Table 1) continued to be seen in the Johns Hopkins Hospital system (Baltimore, MD, USA) through October 2021 (Appendix Figure 1). Enterovirus/rhinovirus were detectable throughout the pandemic (*1*,*2*), but their positivity markedly increased to reach 20.7% (of all samples tested for enterovirus/rhinovirus) in October 2021, surpassing all other respiratory viruses (Appendix Figure 2) (*2*).

Enterovirus-D68 (EV-D68) was associated with a large outbreak of respiratory disease in children in North America in 2014 and was subsequently linked to the occurrence of acute flaccid myelitis (AFM) (*3*). After the 2014 outbreak, active surveillance of EV-D68 was implemented in many countries in Asia, Europe, Africa, and the Americas. Data obtained through surveillance during 2014–2018 suggested a biennial circulation cycle in Europe and North America (*4*,*5*). However, despite this expected biennial pattern, EV-D68 detection in 2020 was lower than anticipated, and limited cases were detected in the United States (*6*). This change in the circulation of EV-D68 in 2020 might have been secondary to the widespread mitigation measures for COVID-19. Of note, a recent study from 8 countries in Europe reported a rapid increase in EV-D68 infections during July 31–October 14, 2021, which coincided with a period of relaxed COVID-19 mitigation measures (*7*).

For this study, we collected samples positive for enterovirus/rhinovirus after the standard-of-care diagnosis at the Johns Hopkins Medical Microbiology Laboratory during September–October 2021 (Figure; Appendix). Research was conducted under Johns Hopkins Institutional Review Board protocol IRB00221396 with a waiver of consent. Remnant nasopharyngeal clinical specimens from patients that tested positive for enterovirus/rhinovirus during September–October 2021 were retrieved for the study. Genomes were made publicly available in GenBank (accession nos. OL826825–36).

**Figure:**
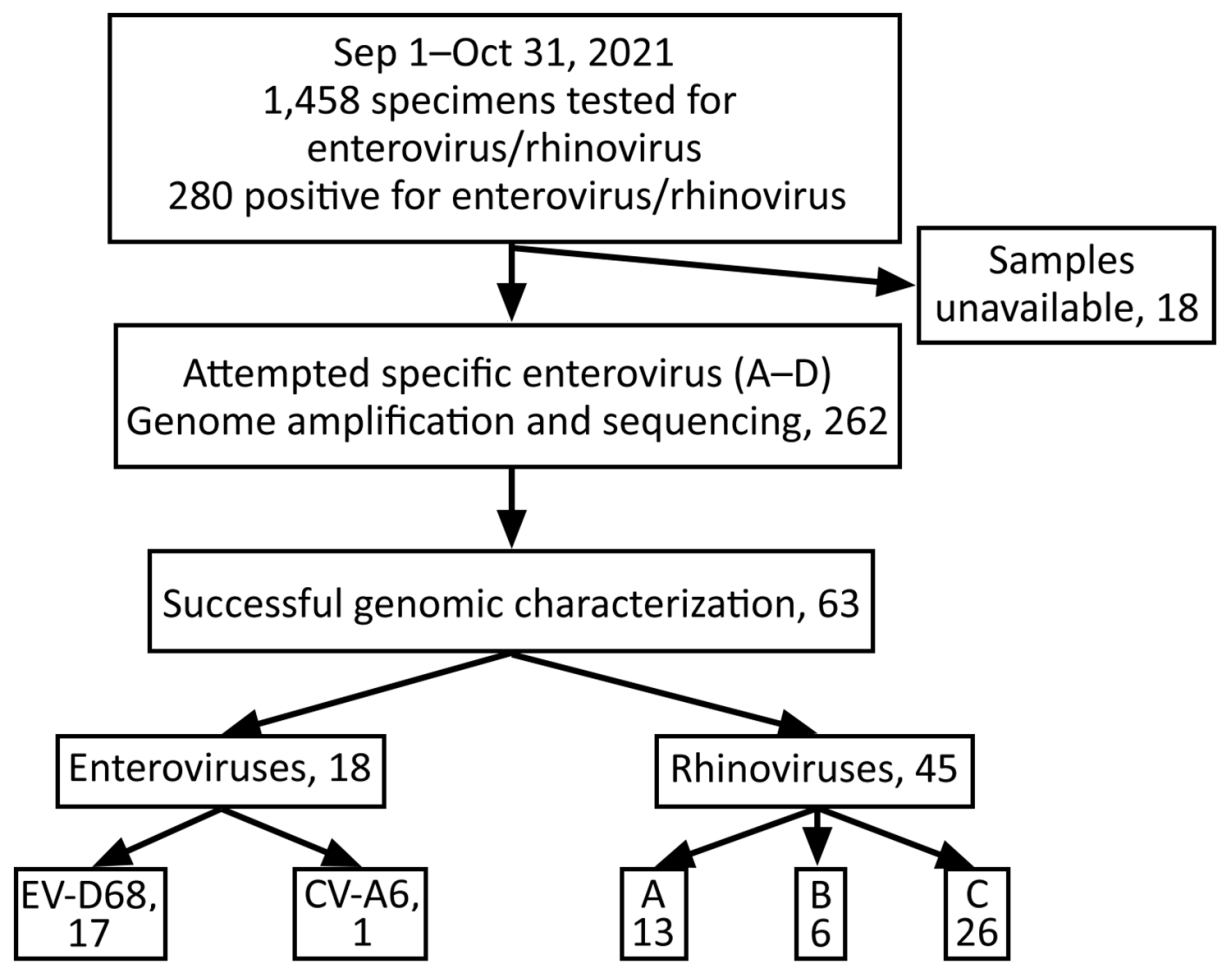
Flowchart of patients and specimens in study of circulation of EV-D68 during period of increased influenza-like illness, Maryland, USA, 2021. CV-A6, coxsackievirus A6; EV-D68, enterovirus D68.

We employed an optimized typing approach by using Nanopore next-generation sequencing (NGS) to characterize the enterovirus types (September–October 2021) associated with the increase in influenza-like illness. In brief, we used primers specific for enterovirus species A–D to amplify a ≈4,500-nt fragment that covers the whole P1 region (about half of the genome) (*8*) and then performed sequencing (Appendix). Of 280 enterovirus/rhinovirus-positive samples, we collected 262 for genotyping (Figure). We detected enterovirus in 28.6% of the 63 successfully sequenced samples (18/63); 94.4% (17/18) were EV-D68 and 5.6% (1/18) were coxsackievirus A6 (CV-A6). Even though the primers used for amplification were specific for enteroviruses, rhinoviruses were characterized in 45 of the 63 samples; those rhinoviruses consisted primarily of species C (26/45), followed by A (13/45) and B (6/45). 

The whole cohort of patients positive for enterovirus/rhinovirus during September–October 2021 ranged in age from <1 year to >90 years; mean age was 16.7 years and median age 5 years. The male:female ratio was 1:1. On the other hand, the median age of EV-D68–positive patients was 2 years, and the male:female ratio was 1:3 (Appendix Table 2). EV-D68 was detected in 15/168 (8.9%) pediatric specimens positive for enterovirus/rhinovirus during the study time frame. Symptoms or signs of viral or respiratory illness were reported in all pediatric patients with EV-D68 (N = 15) (Appendix Table 2), and 5 patients (33.3%) were admitted and required supplemental oxygen, admission to the intensive care unit, or both. No neurologic complications including AFM were observed (Appendix Table 2). Of note, no AFM cases were diagnosed at Johns Hopkins Hospital during the study time frame. Most cases of enterovirus were detected in residents of the city of Baltimore (11/17). A total of 12 EV-D68 sequences, subclade B3, had a complete 5′ half of the genome (3000–4200 bp). EV-D68 genomes clustered with strains detected in 2019 from several countries in Europe (Appendix Figure 3).

We report a predominance of EV-D68 among the circulating enteroviruses during the same period in which enterovirus/rhinovirus positivity increased in this hospital system (*2*). The predominance of EV-D68 in our study (27% of total enterovirus/rhinovirus-typed genomes) was higher than the 0.4% and 3.6% observed in 2019 and 2020 in the United States (*6*) and comparable to the 24.3% reported before the COVID-19 pandemic in 2018 (*6*).

The EV-D68 strains detected belong to the B3 subclade, which had not been reported from the United States since 2018 (*6*) but was detected in Europe in 2019 (*9*). The strains we detected form a distinct cluster within the B3 subclade that circulated in 2018 in Europe and the United States but seem very close to those characterized in Europe in 2019. Nevertheless, it was reported that strains circulating in Europe in 2019 are common ancestors of strains detected in the United States in 2018 (*9*). That report might explain why the strains we identified are more closely related to subclade B3 from the United States than to those from Europe in 2018.

Appendix Additional information about circulation of enterovirus D68 during period of increased influenza-like illness, Maryland, USA, 2021.
