# Supplementary material for: Circulation of Enterovirus D68 during Period of Increased Influenza-Like Illness, Maryland, USA, 2021
Source: Emerg Infect Dis. 2022 Jul;28(7):1525–7. doi: 10.3201/eid2807.212603 (PMC9239864; doi:10.3201/eid2807.212603)
Supplement: Appendix — Additional information about circulation of enterovirus D68 during period of increased influenza-like illness, Maryland, USA, 2021. [file 21-2603-Techapp-s1.pdf]

**Article DOI:** https://doi.org/10.3201/eid2807.212603

# Circulation of Enterovirus D68 during Period of Increased Influenza-Like Illness, Maryland, USA, 2021

## Appendix

### Patients' Clinical Data

Demographic and clinical data were collected through bulk query from the Johns Hopkins Hospital electronic medical record system. Influenza-like illness encounters were defined based on the following criteria: all emergency department (ED) visits (regardless of age across the entire system) where: (1) patients presented with a chief report of upper respiratory infection (URI), pneumonia, influenza, flu-like symptoms, cough, or fever and sore throat (2) ED diagnosis of B97.89 (other viral agents as the cause of diseases classified elsewhere). Diagnosis codes are detailed in Appendix Table 1. Clinical data presented in Appendix Table 2 were collected by detailed manual chart reviews from the electronic patients' records.

### Specimens and Diagnostic Testing

The Johns Hopkins Microbiology laboratory serves a wide geographic area that includes Baltimore, Virginia, and Washington, DC. Testing for enterovirus/rhinovirus is performed as a part of multiplex respiratory viral panels using the GenMark ePlex RP1 and RP2. These panels do not differentiate between enteroviruses and rhinoviruses (Figure, https://wwwnc.cdc.gov/EID/article/28/7/21-2603-F1.htm) and detect Adenovirus, HCoV-229E, HCoV-HKU1, HCoV-NL63, HCoV-OC43, human Metapneumovirus (HMPV), enterovirus/rhinovirus, influenza A/A H1/A H1–2009/A H3, influenza B, parainfluenza (HPIV)1–4, RSV A, RSV B, *Chlamydia pneumoniae*, and *Mycoplasma pneumoniae* in addition to SARS-COV-2 (in RP2 only) (*1*,*2*).

## Genomic Sequencing for Genotyping

RNA was extracted from 300µL of clinical specimens using the Chemagic 360 system (Perkin Elmer) according to the manufacturer's specifications. RNA was eluted with 60µl nuclease-free water and stored at −80°C until use. cDNA synthesis has been performed using RevertAid First Strand cDNA Synthesis Kit (ThermoFisher Scientific) following the manufacturer's protocol. The amplification of the 5′ half of the genome (~4500 nt) was performed as previously described (*3*). PCR products were barcoded using the Native barcoding genomic DNA kit (EXP-NBD196) according to the manufacturer's instructions and sequenced using R9.4.1 flowcells on a GridION (Oxford Nanopore Technologies). For the FASTQ files analysis, low-quality reads were filtered, and adapters trimmed with Trimmomatic. Denovo assembly was performed using metaSPAdes. Identification of the enterovirus types was carried out with the RIVM genotyping tool (http://www.rivm.nl/mpf/enterovirus/typingtool/). Sequence alignment and phylogeny were performed using MEGA 7.0. The robustness of the ML tree was assessed by bootstrap analyses of 1,000 replicates and the Fermon strain, collected in 1962 was used to root the tree. The evolutionary distances were derived using the Tamura 3 parameter method. BLAST analysis showed >99% homology with strains detected in Europe in 2019 and with a bootstrap of 98%.

### References

1. Jarrett J, Uhteg K, Forman MS, Hanlon A, Vargas C, Carroll KC, et al. Clinical performance of the GenMark Dx ePlex respiratory pathogen panels for upper and lower respiratory tract infections. J Clin Virol. 2021;135:104737. PubMed https://doi.org/10.1016/j.jcv.2021.104737

2. Uhteg K, Amadi A, Forman M, Mostafa HH (2021) Circulation of non- SARS-CoV-2 respiratory pathogens and coinfection with SARS-CoV-2 amid the COVID-19 pandemic. Open Forum Infect Dis. 2021;9:ofab618. **PMID 35211632**

3. Joffret ML, Polston PM, Razafindratsimandresy R, Bessaud M, Heraud JM, Delpeyroux F. Whole genome sequencing of enteroviruses species A to D by high-throughput sequencing: application for viral mixtures. Front Microbiol. 2018;9:2339. PubMed https://doi.org/10.3389/fmicb.2018.02339

4. Jones GF, Fabre V, Hinson J, Levin S, Toerper M, Townsend J, et al. Improving antimicrobial prescribing for upper respiratory infections in the emergency department: Implementation of peer comparison with behavioral feedback. Antimicrobial Stewardship & Healthcare Epidemiology. 2021;1:e70. https://doi.org/10.1017/ash.2021.240

**Appendix Table 1.** Diagnosis codes used to categorize influenza-like illness encounters (*4*)

| Code | Diagnosis |
|---|---|
| B97.89 | Other viral agents as the cause of diseases classified elsewhere |
| H66.9 | Otitis media, unspecified |
| J06.9 | Acute upper respiratory infection, unspecified |
| J00 | Acute nasopharyngitis; common cold |
| J01.9 | Acute sinusitis, unspecified |
| J09.X | Influenza due to identified novel influenza A viruses |
| J10.0 | Influenza due to identified novel influenza A viruses |
| J10.1 | Influenza due to other identified influenza virus with other respiratory manifestations |
| J10.2 | Influenza due to other identified influenza virus with gastrointestinal manifestations |
| J10.8 | Influenza due to other identified influenza virus with other manifestations |
| J11 | Influenza due to unidentified influenza virus |
| J12.89 | Other viral pneumonia |
| J12.9 | Viral pneumonia, unspecified |
| J18 | Pneumonia, unspecified organism |
| J20.9 | Acute bronchitis, unspecified |
| J40 | Bronchitis, not specified as acute or chronic |
| R05 | Cough |
| R50.9 | Fever, unspecified |
| J22 | Unspecified acute lower respiratory infection |
| B34[0–9] | Viral infection of unspecified site [adenovirus, enterovirus, coronavirus, parvovirus, papovavirus, other viral infections |
| U07.1 | COVID-19 |
| R68.89 | Diagnosis reported 'Flu-like symptoms' or 'Influenza-like symptom |

**Appendix Table 2.** Clinical and demographic characters of enterovirus infected patients in September and October 2021

| Genome ID | Enterovirus | Age range, y | Gender | Respiratory/viral Complaint | Admitted | ICU | Supplemental Oxygen |
|---|---|---|---|---|---|---|---|
| JH-EV-0001/2021 | EV-D68 | <1 | Female | Breathing problem | Yes | Yes | Yes |
| JH-EV-0002/2021 | EV-D68 | 45–48 | Male | | | | |
| JH-EV-0003/2021 | EV-D68 | <1 | Female | | Yes | Yes | Yes |
| JH-EV-0004/2021 | EV-D68 | 8–10 | Male | Viral illness | Yes | Yes | Yes |
| JH-EV-0005/2021 | EV-D68 | 23–25 | Female | | | | |

| Genome ID | Enterovirus | Age range, y | Gender | Respiratory/viral Complaint | Admitted | ICU | Supplemental Oxygen |
|---|---|---|---|---|---|---|---|
| JH-EV-0006/2021 | EV-D68 | 1–3 | Male | Rhinorrhea | | | |
| JH-EV-0007/2021 | EV-D68 | <1 | Male | Cough, Bronchiolitis, Acute respiratory distress | Yes | | Yes |
| JH-EV-0008/2021 | EV-D68 | 4–6 | Female | Fever, loss of appetite, cough, runny nose | | | |
| JH-EV-0009/2021 | EV-D68 | <1 | Female | Fever, irritability, congestion, rhinorrhea, sneezing, cough | | | |
| JH-EV-0010/2021 | EV-D68 | 1–3 | Male | Cough, rash | | | |
| JH-EV-0011/2021 | EV-D68 | 1–3 | Female | Respiratory distress, viral pneumonia, enterovirus bronchiolitis | Yes | Yes | Yes |
| JH-EV-0012/2021 | EV-D68 | 1–3 | Male | Cough and nasal congestion | | | |
| JH-EV-0013/2021 | EV-D68 | 1–3 | Male | Fever, productive cough, runny nose | | | |
| JH-EV-0014/2021 | EV-D68 | <1 | Female | Nasal congestion, cough | | | |
| JH-EV-0015/2021 | EV-D68 | 1–3 | Male | Congestion and cough | | | |
| JH-EV-0016/2021 | EV-D68 | 1–3 | Male | Cough, rhinorrhea, fever, respiratory distress, pneumonia | Yes | Yes | Yes |
| JH-EV-0017/2021 | EV-D68 | 1–3 | Female | Nasal congestion and fever | | | |
| JH-EV-0018/2021 | CV-A6 | 32–35 | Male | Rash | | | |

*ICU, intensive care unit.

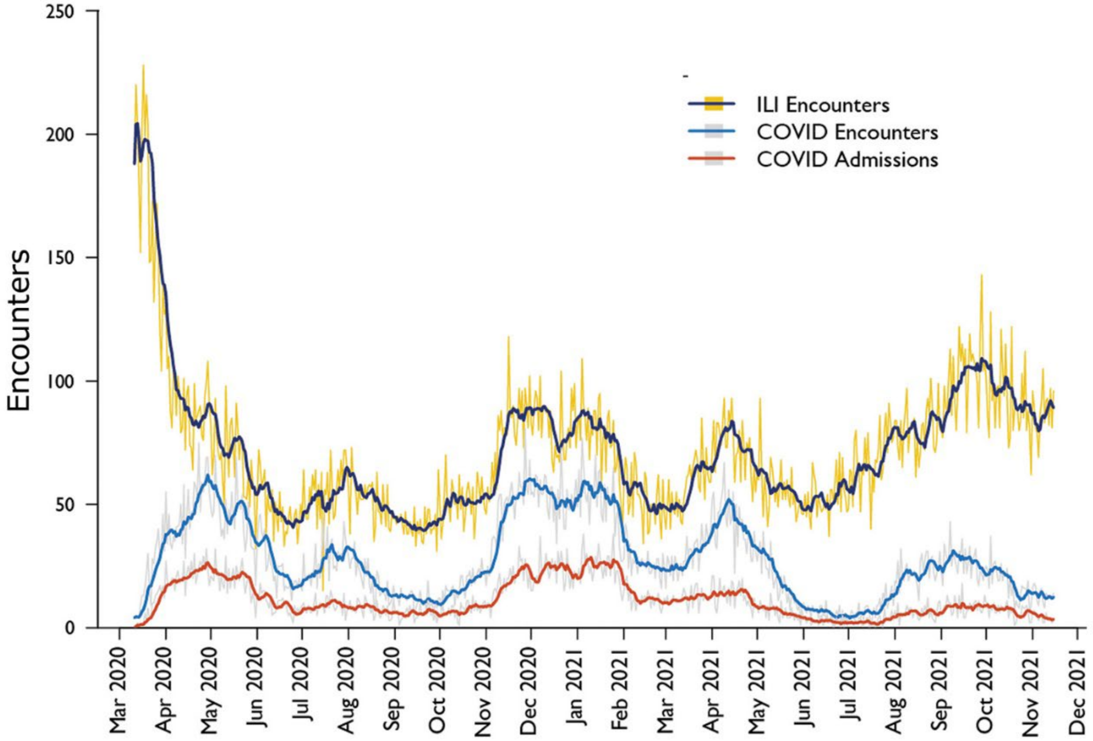


**Appendix Figure 1.** Encounters of influenza-like illness (ILI), COVID-19 encounters, and admissions since the beginning of the COVID-19 pandemic at Johns Hopkins Hospital system.

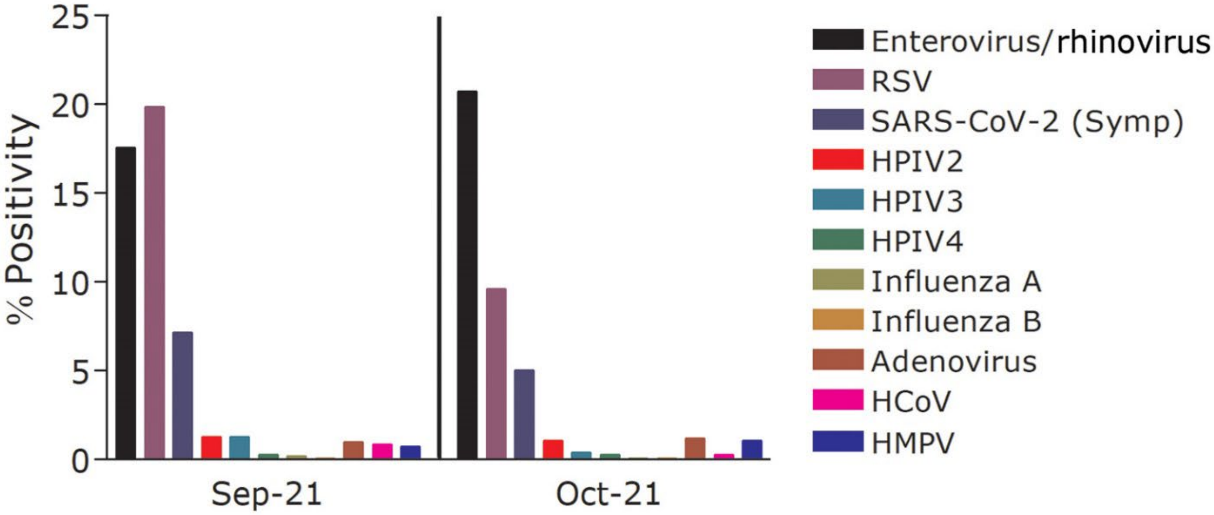


**Appendix Figure 2.** Positivity of respiratory viral targets at Johns Hopkins Medical Microbiology laboratory in September and October 2021. HPIV (human parainfluenza virus), RSV (respiratory syncytial virus), HMPV (human metapneumovirus), SARS-CoV-2 (symp) indicates symptomatic testing positivity.

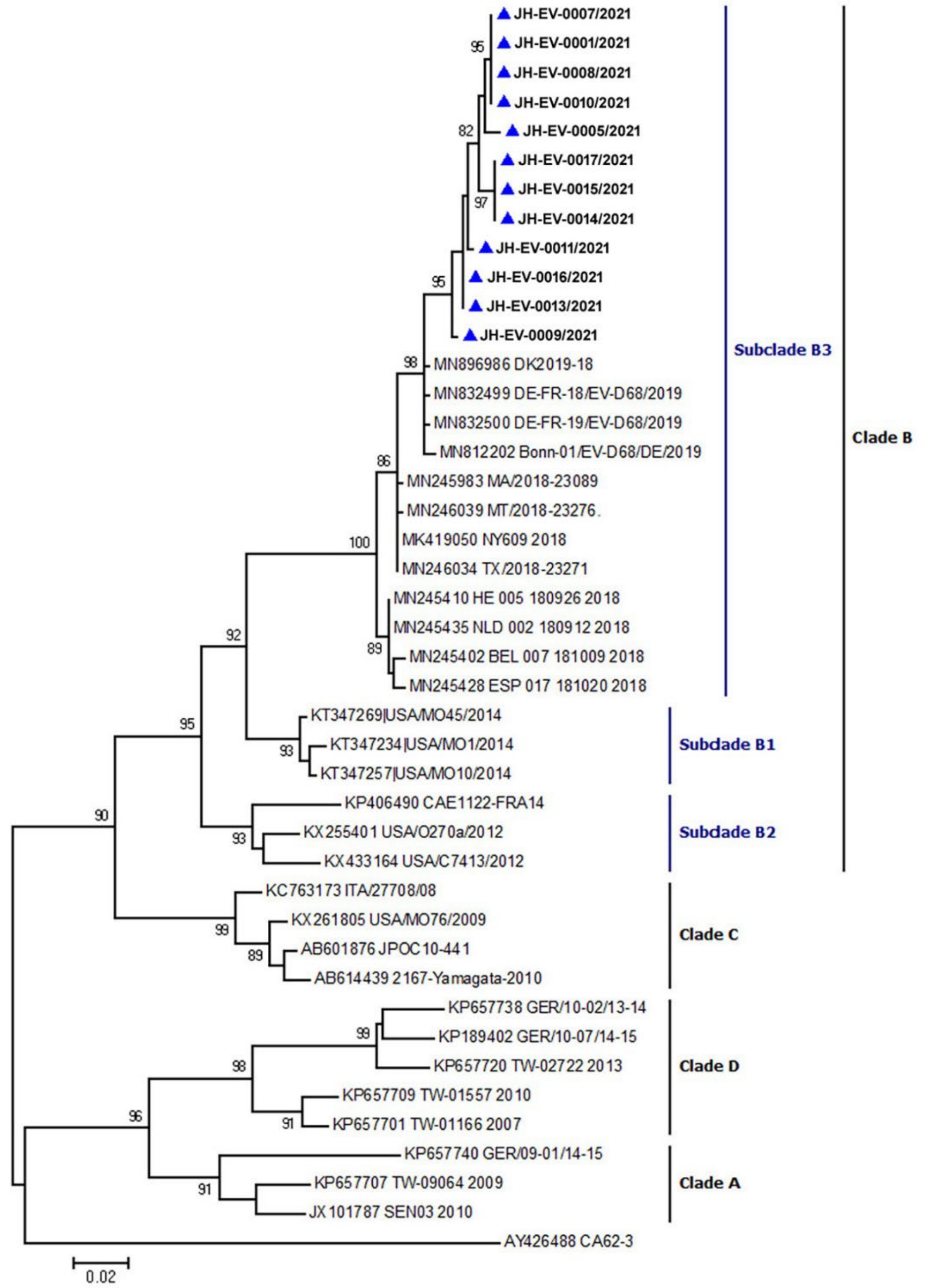


**Appendix Figure 3.** Phylogenetic relationships of EV-D68 strains identified from the Johns Hopkins Medical Microbiology laboratory between September and October 2021 (marked with blue triangles). The phylogenetic tree constructed on complete 5′ half of the genome of EV-D68 strains was generated using the Maximum Likelihood method based on the using the Tamura 3 parameter method in MEGA7. The phylogenetic tree is rooted by the oldest EV-D68 sequence in GenBank, the Fermon strain. We performed 1,000 bootstrap replicates to determine the consensus tree; support for nodes present in >70% of the trees are annotated.
